# The relation between deoxycytidine kinase activity and the radiosensitising effect of gemcitabine in eight different human tumour cell lines

**DOI:** 10.1186/1471-2407-6-142

**Published:** 2006-05-30

**Authors:** Bea Pauwels, Annelies EC Korst, Greet GO Pattyn, Hilde AJ Lambrechts, Juliette AE Kamphuis, Christel MJ De Pooter, Godefridus J Peters, Filip Lardon, Jan B Vermorken

**Affiliations:** 1 Laboratory of Cancer Research and Clinical Oncology, Department of Medical Oncology, University of Antwerp (UA/UZA), Wilrijk, Belgium; 2 Department Medical Oncology, VU University Medical Center, Amsterdam, The Netherlands; 3 Department of Radiotherapy, St Augustinus Hospital, Wilrijk, Belgium

## Abstract

**Background:**

Gemcitabine (dFdC) is an active antitumour agent with radiosensitising properties, shown both in preclinical and clinical studies. In the present study, the relation between deoxycytidine kinase (dCK) activity and the radiosensitising effect of gemcitabine was investigated in eight different human tumour cell lines.

**Methods:**

Tumour cells were treated with dFdC (0–100 nM) for 24 h prior to radiotherapy (RT) (γ-Co^60^, 0–6 Gy, room temperature). Cell survival was determined 7, 8, or 9 days after RT by the sulforhodamine B test. dCK activity of the cells was determined by an enzyme activity assay.

**Results:**

A clear concentration-dependent radiosensitising effect of dFdC was observed in all cell lines. The degree of radiosensitisation was also cell line dependent and seemed to correlate with the sensitivity of the cell line to the cytotoxic effect of dFdC. The dCK activity of our cell lines varied considerably and differed up to three fold from 5 to 15 pmol/h/mg protein between the tested cell lines. In this range dCK activity was only weakly related to radiosensitisation (correlation coefficient 0.62, p = 0.11).

**Conclusion:**

Gemcitabine needs to be metabolised to the active nucleotide in order to radiosensitise the cells. Since dFdCTP accumulation and incorporation into DNA are concentration dependent, the degree of radiosensitisation seems to be related to the extent of dFdCTP incorporated into DNA required to inhibit DNA repair. The activity of dCK does not seem to be the most important factor, but is clearly a major factor. Other partners of the intracellular metabolism of gemcitabine in relation to the cell cycle effects and DNA repair could be more responsible for the radiosensitising effect than dCK activity.

## Background

Gemcitabine (2',2'-difluorodeoxycytidine, dFdC) is a synthetic pyrimidine nucleoside analogue that has a structure very similar to that of deoxycytidine and cytosine arabinoside (Ara-C) [1]. In clinical use, gemcitabine is active against a variety of solid tumours such as cancers of the pancreas, lung, head and neck, bladder, breast, and ovary. It is activated intracellularly by deoxycytidine kinase (dCK), which adds a phosphate group to the 5' position of the deoxyribose group. The diphosphate (dFdCDP) and triphosphate (dFdCTP) forms of the drug play an important role in the cytotoxic effect: dFdCDP is an inhibitor of ribonucleotide reductase, while dFdCTP is incorporated into DNA, both leading to the inhibition of DNA synthesis. At the same time, gemcitabine has several self-potentiation mechanisms that serve to increase intracellular levels of the active metabolite and increase cytotoxicity [1,2]

In addition to its cytotoxic effect, gemcitabine is a potent radiosensitiser when used in rodent and human tumour cells, including pancreatic tumours, non-small cell lung cancer, head and neck cancer, colorectal, breast, ovarian and bladder cancer [3-14]. Gemcitabine can lead to a radiosensitising effect in both monolayer and spheroid glioblastoma cultures [15]. Recent *in vivo *studies have confirmed these observations and have shown significant tumour growth delay with the combination of gemcitabine and ionising radiation in animal models [4,5,16]. These results have prompted a variety of clinical trials using gemcitabine as a radiosensitiser [17-31]. The exact mechanism of radiosensitisation and most important factors are still not known yet. Although the effects of gemcitabine on cell cycle redistribution and deoxynucleotide triphosphate (dNTP) pools may contribute to, or even be necessary for, gemcitabine-mediated radiosensitisation [7,10,13], they do not ultimately determine whether or not gemcitabine treatment is going to result in enhanced radiosensitivity. For example, the role of dATP depletion could not be confirmed in a study using cells with different repair defects [32]. Wachters et al [33] have shown that gemcitabine can sensitise cells to radiation by specific interference with the homologous repair (HR) pathway. Others have suggested that gemcitabine in combination with radiotherapy is compromised by mismatch repair (MMR), because recovery from gemcitabine treatment is facilitated by, although not dependent on MMR proficiency [34,35]

As a prodrug, gemcitabine requires intracellular phosphorylation to its active triphosphate form by dCK and to a lesser extent by thymidine kinase (TK2) [36] to exhibit biological activity. Acquired resistance to gemcitabine has been associated with deficiency of dCK [37-40] It has been shown that the expression of dCK at mRNA, protein and activity level in cancer cell lines of different origin were closely related and a correlation between the sensitivity to gemcitabine and the activity of dCK was observed [41]. It has also been shown that sensitivity to nucleoside analogues could be restored by transfection of a wild type dCK cDNA [42-44]. The critical role of dCK in gemcitabine's radiosensitisation is not known. Previously a significant relationship between dCK activity and sensitivity of various xenografts to gemcitabine was described [41] while accumulation of the triphosphate, dFdCTP, was related to sensitivity to gemcitabine also [45]. Earlier data with a gemcitabine resistant cell line, i.e. lacking dCK, showed that these cells required a high concentration (50–100 μM) of gemcitabine in order to get radiosensitisation [46]. However, because radiosensitisation *in vitro *increases with the drug concentration [12,47])., it could be that the radiosensitising effect of gemcitabine also depends on the rate of drug phosphorylation which in turn depends on dCK activity [8] and drug concentration [12,13,37,45,48]). Therefore, it has be hypothesized that dCK activity could be an important factor for the radiosensitising effect of gemcitabine.

The purpose of the present study was to further substantiate the role of dCK in gemcitabine's radiosensitisation. Most studies investigating the radiosensitising effect of gemcitabine are limited in cell types and only a few concentrations of gemcitabine are used. In this study, the radiosensitising effect of gemcitabine was investigated in eight human tumour cell lines, originating from different tissues, using a range of gemcitabine concentrations. Within that context, the role of dCK was further explored.

## Methods

### Chemicals and reagents

Dulbecco's Modified Eagle's Medium (DMEM), RPMI, Medium 199, fetal calf serum and the medium supplements L-glutamine and sodium pyruvate were all purchased from Invitrogen (Merelbeke, Belgium). Sulforhodamine B was obtained from ICN (Asse, Belgium). Gemcitabine was purchased from Eli Lilly (Indianapolis, USA).

### Cell lines

The cell lines used in this study were human tumour cells differing in p53 status originating from different tissues: ECV304 (mt-p53) a human epidermoid bladder cancer cell line, H292 (wt-p53) a human mucoepidermoid lung cancer cell line, A549 (wt-p53) a human squamous lung cancer cell line, MCF-7 (wt-p53) a mammary carcinoma cell line, HT-29 (mt-p53) a colon adenocarcinoma cell line, Panc-1 (mt-p53) a human pancreatic epitheloid cell line, CAL-27 (mt-p53) a human squamous cell carcinoma cell line of the tongue and FaDu (mt-p53) a human squamous cell carcinoma cell line of the pharynx. H292 and A549 were cultured in RPMI-1640 medium, supplemented with glutamine, sodium pyruvate and 10% fetal calf serum. ECV304 was cultured in Medium-199 supplemented with 10% fetal calf serum. MCF-7, HT-29, CAL-27 and FaDu were cultured in DMEM medium, supplemented with glutamine and 10% fetal calf serum. Cultures were maintained in exponential growth in a humidified atmosphere at 37°C under 5% CO_2_/95% air.

### Cell survival after treatment with gemcitabine and radiation

The sulforhodamine B (SRB) test is a suitable test system for in vitro radiosensitivity testing, which in the presently used cell lines has shown to be comparable in outcome with the clonogenic assay, when cells are allowed to undergo at least 6 doubling times after radiation treatment [49]. Therefore, in our experiments, ECV304, H292, A549, MCF-7, HT-29 and FaDu cells were incubated for 7 days, CAL-27 cells for 8 days and Panc-1 cells for 9 days after radiation treatment, before determination of the survival by the SRB assay. Optimal seeding densities were determined for each cell line to assure exponential growth during the assay.

Cells were harvested from exponential phase cultures by trypsinisation, counted and plated in 48-well plates. Following plating and a 24 h recovery period, cells were treated with gemcitabine (0–100 nM) dissolved in phosphate buffered saline (PBS) during 24 h immediately followed by radiation. PBS was added to control cells. Each concentration was tested six times within the same experiment. After irradiation at room temperature over a dose range of 0–6 Gy, using a ^60^Co source (Alcyon, St Augustinus hospital, Antwerp), cells were washed with drug free medium. After 7, 8 or 9 days, the survival was determined by the SRB assay. For determination of cell survival after treatment with gemcitabine alone, the SRB assay was performed 4 days after the start of treatment.

The SRB assay was performed according to the method of Skehan and colleagues and Papazisis and colleagues, with minor modifications [50,51] Culture medium was aspirated prior to fixation of the cells by addition of 200 μl 10% cold trichloroacetic acid. After 1 h incubation at 4°C, cells were washed 5 times with deionised water. Then the cells were stained with 200 μl 0.1% SRB dissolved in 1% acetic acid for at least 15 minutes and subsequently washed 4 times with 1% acetic acid to remove unbound stain. The plates were left to dry at room temperature and bound protein stain was solubilised with 200 μl 10 mM unbuffered TRIS base (tris(hydroxymethyl)aminomethane) and transferred to 96 wells plates for reading the optical density at 540 nm (Biorad 550 microplate reader, Nazareth, Belgium).

### dCK enzyme activity assays

To determine a possible correlation between dCK activity and the radiosensitising effect of gemcitabine, cells were harvested as described previously [41], and pellets were stored at -80°C until analysis. 25.10^6 ^cells were used in order to be able to measure enzyme activity in a linear range for time and protein. dCK was determined essentially as described previously [41]. To measure dCK selectively and bypass TK2 mediated phosphorylation of deoxycytidine, we used radiolabeled chlorodeoxyadenosine ([^3^H]CdA) as the substrate [52], which is not activated by TK2. Enzyme activities were expressed as nmol product per h per mg protein (nmol/h/mg protein).

### Statistical methods

The survivals were calculated by: mean optical density (OD) of treated cells/mean OD of control cells × 100%. The radiation survival curves were fitted according to the linear-quadratic model: surviving fraction = exp(- αD – βD^2^), using Winnonlin (Pharsight, USA).

The following parameters were calculated: ID50, the radiation dose causing 50% growth inhibition and IC50, the concentration gemcitabine causing 50% growth inhibition. The radiosensitising effect was represented by the dose enhancement factor (DEF): ID50(-dFdC)/ID50(+dFdC).

Unless otherwise indicated, all data are presented as the mean ± standard deviation. All experiments were performed at least three times. A two-sample t-test and one-way ANOVA analysis was used to determine significance between ID50 values and DEFs.

Radiosensitisation can be defined as a synergistic interaction between gemcitabine and radiation. For the determination of synergism, the combination index (CI) was calculated by the Chou-Talalay equation [47,53,54], using CalcuSyn (Biosoft, USA, UK). The general equation for the classic isobologram is given by:



where (D_x_)_1 _and (D_x_)_2 _in the denominators are the doses (or concentrations) for D_1 _(gemcitabine) and D_2 _(radiation) alone that give x% inhibition, whereas (D)_1 _and (D)_2 _in the nominators are the doses of gemcitabine and radiation in combination that also inhibit x% (i.e., isoeffect).

The (D_x_)_1 _or (D_x_)_2 _(for gemcitabine and radiation) can be readily calculated from the median-effect equation of Chou:



Where f_a _is the fraction affected and D_m _is the median-effect dose (ID50 or IC50) that is obtained from the anti-log of the X-intercept of the median effect plot, X-log (D) versus Y = log [f_a_/(1 - f_a_] or D_m _= 10^-(Y-intercept)/m^, and m is the slope of the median effect plot.

For conservative mutually nonexclusive isobolograms of two agents, a third term,



is added. The third term is usually omitted, when the mutually exclusive (α = 0) assumption or classic isobologram is used [53,55] Data in table 2 are based on the assumption that α = 0.

A CI value between 0.9 and 1.1 indicates only additivity. Moderate synergism is depicted by CI values between 0.7 and 0.9, synergism by CI values below 0.7.

## Results

### Radiosensitisation by gemcitabine

**Figure 1:**
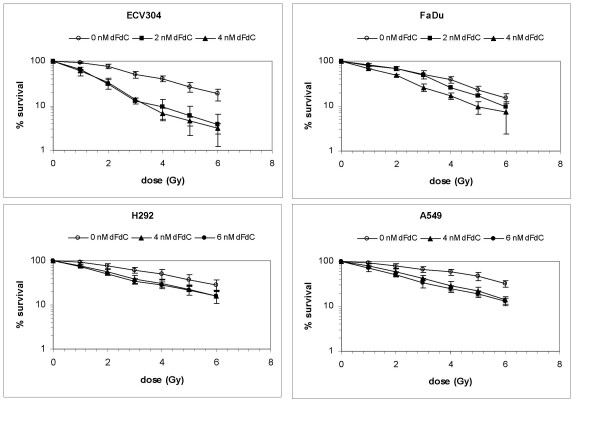
*Radiation dose response curves*. Radiation dose responsecurves of ECV304, H292, A549, and FaDu cells after treatment with gemcitabine (dFdC) during 24 h immediately before radiation (RT) (survival ± S.D.)

**Figure 2:**
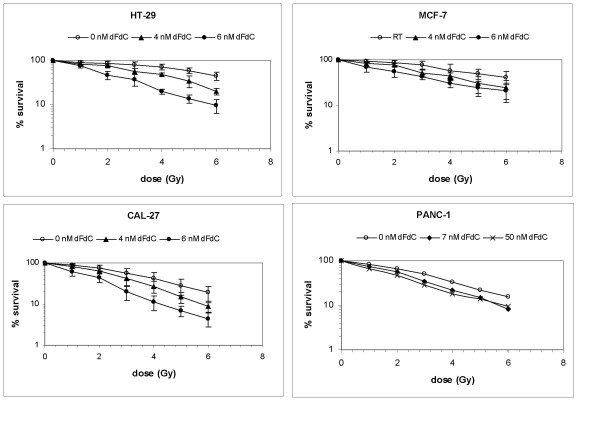
*Radiation dose response curves*. Radiation dose responsecurves of HT-29, MCF-7, CAL-27 and Panc-1 cells after treatment with gemcitabine (dFdC) during 24 h immediately before radiation (RT) (survival ± S.D.)

A clear concentration-dependent radiosensitising effect of gemcitabine was observed in ECV304, FaDu, H292, A549, CAL-27, Panc-1, MCF-7 and HT-29 cells (Figure 1 and 2). ID50 values and DEFs for the different gemcitabine concentrations are summarised in Table 1.

The degree of radiosensitisation seemed to be cell line dependent. For ECV304 and MCF-7 cells, the DEF after treatment with 1 nM gemcitabine was 1.37, and 1.24, respectively, while this concentration had no radiosensitising effect in FaDu, H292, A549, CAL-27 and HT-29 cells with DEF values around 1. In Panc-1 cells, much higher concentrations (15–100 nM) of gemcitabine were required to obtain a radiosensitising effect than in the other cell lines (2–8 nM). Panc-1 cells were less sensitive for the cytotoxic effect of gemcitabine alone as depicted by the IC50 values in Table 1. The radiosensitising effect seemed to correlate with the sensitivity of the cell line to the cytotoxicity effects of gemcitabine (correlation coefficient for mean IC50 and mean DEF: -0.82, p = 0.013).

**Figure 3:**
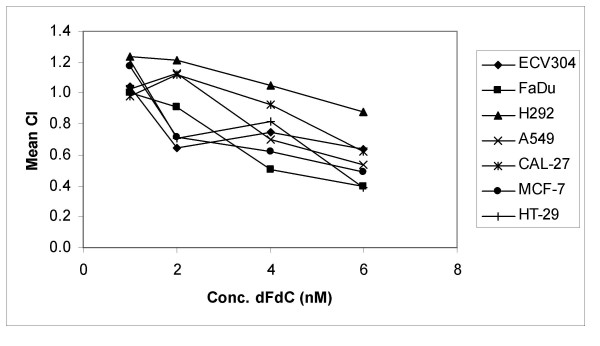
*Relationship between gemcitabine concentration and the combination index*. Relationship between the concentration of gemcitabine (dFdC) and the mean combination index (CI) for ECV304, FaDu, H292, A549, CAL-27, MCF-7 and HT-29 cells. The cells were treated during 24 h with gemcitabine immediately before radiotherapy (0–8 Gy). A CI value between 0.9 and 1.1 indicates only additivity. Moderate synergism is depicted by CI values between 0.7 and 0.9, synergism by CI values below 0.7.

The CI analysis showed that after treatment during 24 h with gemcitabine immediately before radiation treatment, there is synergism in ECV304 with gemcitabine concentrations of 2 nM or higher (CI ≤ 0.65), with concentrations of 4 nM or higher in A549 (CI ≤ 0.70), MCF-7 (CI ≤ 0.62) and FaDu (CI ≤ 0.50) and with concentrations of 6 nM and higher in HT-29 cells (CI ≤ 0.39). Only moderate synergism was observed with 6 nM or higher in H292 (CI ≤ 0.88) and CAL-27 (CI ≤ 0.72). In Panc-1 cells, concentrations of 7 nM and higher resulted in a moderate to synergistic interaction (CI ≤ 0.79) (Figure 3).

### dCK

**Figure 4:**
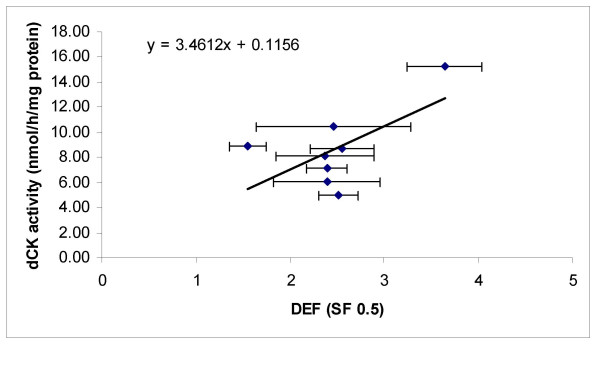
*Relationship between deoxycytidine kinase activity and the radiosensitising effect of gemcitabine*. Relationship between deoxycytidine kinase (dCK) activity and the radiosensitising effect of gemcitabine represented by the dose enhancement factor (DEF) for the different human tumour cells. (SF = surviving fraction)

Since our cell lines had a normal sensitivity range (nM range) and had a DEF comparable to high and low values in literature, we measured dCK activity in order to determine whether there would be relation between DEF and dCK activity. Table 2 represents the dCK activity per cell line. The dCK activity varied considerably from cell line to cell line and differed up to three fold from 5 to 15 pmol/h/mg protein between the tested cell lines. In this range dCK activity was weakly related to DEF (correlation coefficient = 0.62, p = 0.11) (Figure 4).

## Discussion

This is the first study showing a clear concentration-dependent radiosensitising effect of gemcitabine in a large number of tumour cell lines, originating from different tissues. In addition, we found that that there was a weak positive correlation between dCK activity of these cells and the DEF. Probably, more factors, including other partners of the intracellular metabolism, cell cycle effects and DNA repair play a role. This offers an explanation for the variable results in the clinic.

In particular, we investigated the radiosensitising effect in 8 different cell lines, using various concentrations of gemcitabine. Our data demonstrate that gemcitabine increases the radiosensitivity of ECV304, H292, A549, MCF-7, HT-29, CAL-27, Panc-1 and FaDu cells in vitro when the cells are treated for 24 h immediately before radiation. The enhancement is concentration dependent, with an increasing DEF with higher concentrations of gemcitabine.

Our DEFs are comparable to literature data from other cell lines [6-13,46]). In most of these studies, the concentration of gemcitabine needed to achieve such a radiosensitising effect, was higher than in our study. This seems to be dependent on the sensitivity to the single agent gemcitabine of the cell lines studied since in gemcitabine-resistant cell lines even μM concentrations of gemcitabine were required [46]. In addition, this could also be due to differences in DNA repair between the cells. In our experiments, the radiosensitising effect was cell line dependent; for example, higher gemcitabine concentrations were needed to induce radiosensitisation in Panc-1 cells (Table 1).

As mentioned gemcitabine is a prodrug that requires successive intracellular phosphorylations into its active trisphosphate form [56]. The enzyme dCK is required for the first phosphorylation step into dFdCMP, while non-specific kinases are responsible for the further phosphorylation steps. As it has been reported that the level of enzymatic activity of dCK could have a profound influence on cellular resistance to gemcitabine cytotoxicity, the present investigations were also designed to address the relationship between gemcitabine's radiosensitisation and the activity of dCK in various human tumour cell lines. The range of dCK activity found in our cell lines, is in agreement with the range found in other solid tumour cell lines, but lower than in leukemic cell lines [57]. For those cell lines investigated the extent of overall gemcitabine phosphorylation to dFdCTP is related to the drug concentration and the activity of dCK [37,45,57]. However other enzymes also contribute to the overall accumulation and particularly the retention of dFdCTP, such as deoxycytidine deaminase, pyrimidine 5'nucleotidase (5'NT), aspecific nucleotidases and phosphatases, and incorporation into DNA and RNA.

Our results in 8 different human solid tumour cell lines with a varying radiosensitising effect of gemcitabine did not further validate the correlation between dCK activity of the cells and the radiosensitising effect of gemcitabine. Gregoire et al [58] reported a correlation between dCK activity and gemcitabine's radiosensitisation. This correlation held both for dCK mRNA expression and the protein versus radiosensitisation [58]. Contrary to this, we could only show a weak relation between the DEF and dCK activity of eight different tumour cells. Possibly the range of dCK activity in our panel was not large enough. In the xenografts investigated for a relation between dCK activity and gemcitabine's antitumor activity was larger [41].

Several hypotheses have been proposed to explain the radiosensitisation potential of gemcitabine [59]. Of these, most likely, cell cycle synchronization [7,60,61] and DNA repair [32] play an important role in the radiosensitisation. In addition to the intracellular metabolism of gemcitabine, for which dCK is a key factor, intrinsic variation in the cellular response to the analogue after ionising radiation is likely to also play a role in gemcitabine's radiosensitisation. The relative importance of dCK activity among these various parameters is, however, not clear. Possibly the overall rate of initial gemcitabine phosphorylation is also determined by the equilibrium between the activities of dCK and the degrading enzyme, 5'NT, since the ratio dCK/5'NT showed a better correlation with gemcitabine sensitivity than dCK activity alone [62]. Moreover, the final mode of action of gemcitabine of producing a "masked chain termination" after its incorporation into DNA, favours that DNA repair plays an important role with respect to sensitising the tumour cells to radiation.

## Conclusion

Our study suggest that other partners of the intracellular metabolism of gemcitabine in relation to cell cycle effects and DNA repair could be more responsible for the radiosensitising effect of gemcitabine than dCK activity of the cell.

## Competing interests

We have received financial support for doing studies with gemcitabine. Eli Lilly Company is not financing this manuscript.

## Authors' contributions

BP participated in the design of the study, performed cell survival experiments, statistic analysis and drafted the manuscript; AEK, participated in the study design and coordination; GGP and HJL participated in the cell survival experiments and performed cell culture; JAK carried out the dCK activity measurement; CDP was involved in the irradiation experiments; GJP has made substantial contribution to the analysis and interpretation of the data and has been revising the manuscript critically; FL participated in the study design and coordination, helped to draft the manuscript and has been revising the manuscript critically; JBV participated in the coordination of the study, has been involved in drafting the manuscript and revising it critically.

## Pre-publication history

The pre-publication history for this paper can be accessed here:



## Figures and Tables

**Table 1:** IC50, ID50 and DEFs of the different cell lines. Sensitivity of different cell lines for gemcitabine (indicated by IC50 values) and radiotherapy (indicated by ID50 values) alone and the radiosensitising effect of gemcitabine represented by dose enhancement factors (DEF). (mean ± S.D.).

**Cell line**	**IC50 (nM)**	**ID50 (Gy)**	**DEF 1 nM**	**DEF 2 nM**	**DEF 4 nM**	**DEF 6 nM**	**DEF 8 nM**
ECV304	3.05 ± 0.49	3.37 ± 0.40	1.37 ± 0.06	2.41 ± 0.24	3.21 ± 0.25	3.64 ± 0.40	n.a.
FaDu	4.90 ± 0.46	3.09 ± 0.39	1.03 ± 0.04	1.22 ± 0.17	1.80 ± 0.26	2.37 ± 0.52	n.a.
H292	7.99 ± 0.77	4.00 ± 0.86	1.08 ± 0.10	1.23 ± 0.20	1.58 ± 0.22	2.07 ± 0.55	2.46 ± 0.82
A549	9.01 ± 0.89	4.51 ± 0.70	1.00 ± 0.04	1.21 ± 0.17	1.89 ± 0.51	2.39 ± 0.57	2.77 ± 0.26
CAL-27	8.92 ± 1.59	3.52 ± 0.88	1.06 ± 0.07	1.08 ± 0.04	1.49 ± 0.21	2.06 ± 0.06	2.51 ± 0.21
MCF-7	6.94 ± 2.03	4.96 ± 1.27	1.24 ± 0.06	1.45 ± 0.27	1.63 ± 0.13	2.55 ± 0.34	n.a.
HT-29	7.75 ± 1.61	5.64 ± 1.02	1.12 ± 0.21	1.30 ± 0.10	1.62 ± 0.04	2.39 ± 0.21	n.a.

**Cell line**	**IC50 (nM)**	**ID50 (Gy)**	**DEF 7 nM**	**DEF 15 nM**	**DEF 35 nM**	**DEF 50 nM**	**DEF 100 nM**

Panc-1	77.8 ± 9.1	2.92 ± 0.35	1.20 ± 0.10	1.50 ± 0.19	1.55 ± 0.19	1.83 ± 0.20	1.56 ± 0.08

n.a. not analysed

**Table 2:** Deoxycytidine kinase (dCK) activity per cell line.

**Cell line**	**dCK activity (nmol/h/mg protein)**
ECV304	15.29
FaDu	8.12
H292	10.51
A549	6.02
CAL-27	5.02
MCF-7	8.72
HT-29	7.11
Panc-1	8.89
